# How does the e-cigarette industry respond to tax adjustments? Evidence from China

**DOI:** 10.18332/tid/186355

**Published:** 2024-05-09

**Authors:** Rong Zheng, Lingyun Meng, Shidong Su, Mark Goodchild

**Affiliations:** 1 School of International Trade and Economics, University of International Business and Economics, Beijing, China; 2 World Health Organization, Geneva, Switzerland

**Keywords:** e-cigarettes, tax pass-through, TaXSiM model, policy effects

## Abstract

**INTRODUCTION:**

China enacted an excise tax on e-cigarettes in November 2022, which offers a distinctive opportunity to examine the industry's reactions to this fiscal adjustment. This study delves into the industry's pricing strategies following the introduction of the excise tax, facilitating a thorough assessment of the subsequent impact on market dynamics and the government's revenue streams.

**METHODS:**

We developed a TaXSiM model specifically tailored for e-cigarettes in China by integrating the country's e-cigarette tax framework. Our approach involved leveraging market data obtained from a representative product, the RELX Phantom Series, to ensure the model's effectiveness and relevance.

**RESULTS:**

The excise implementation of 2022 significantly heightened the tax burden on e-cigarettes, marking an increase of approximately 150 RMB per device and 19 RMB per cartridge. Despite these financial pressures, electronic cigarette firms exemplified by RELX, strategically endeavored to sustain competitiveness. Their approach involved initially implementing a ‘Razor blade model’ and eventually a ‘comprehensive under-shifting’ strategy, which mitigated the health impact of the tax hike, resulting in a relatively minor decline in sales while amplifying the impact on tax revenue. However, this strategic pricing maneuver came at a cost, as it led to a substantial decrease in profits, and therefore expedited a reshuffling of the industry by compelling smaller brands to leave the market rapidly.

**CONCLUSIONS:**

To effectively curb the use of e-cigarettes through tax policies, it is advisable to relocate the imposition of excise taxes on electronic cigarettes to the retail stage. This shift aims to narrow the scope for industry-level pricing strategies. Furthermore, this approach should be coupled with the introduction of an additional specific tax, strategically crafted to accentuate the health-related benefits associated with the excise taxation on electronic cigarettes.

## INTRODUCTION

Increasing tobacco taxes and raising tobacco product prices are acknowledged as potent measures for curtailing tobacco consumption^1^. However, the tobacco industry has been actively endeavoring to influence tobacco control policies, chiefly through the implementation of intricate pricing strategies^2-4^. These strategies may involve absorbing the increase in tax and avoiding transferring this to consumers through price hikes (a method known as tax under-shifting), which lessens the effect of the policy. Alternatively, they may choose to pass-on the entire tax increase onto consumers or establish a price increase that surpasses the extent of the tax rise (a practice termed as tax over-shifting), thereby amplifying the effect on tax revenues and industry profit^5^. Furthermore, the pricing strategy of the tobacco industry is also influenced by tobacco excise structure^6,7^ and the level of cigarette market competition^8^.

In recent years, there has been a marked increase in the consumption of e-cigarette products^9^, most notably among younger demographics^10^. Existing medical research underscores the potential health ramifications associated with e-cigarettes^11-15^, thereby prompting many governments across the globe to implement taxation policies similar to those applied to traditional cigarettes, to limit public consumption. It is, however, important to note that the e-cigarette industry continues to employ unique pricing strategies designed to undermine the impact of government-imposed taxation. According to a study conducted by Cotti et al.^16^ (2022) using the NielsenIQ Retail Scanner Dataset (NRSD) from 2013 to 2019, nearly 90% of taxes levied on e-cigarettes were passed on to consumer retail prices. Considering the vast range of e-cigarette products, which includes various models and usage modalities, the industry’s primary promotional pricing strategy involves direct price cuts and volume-based pricing incentives^17,18^. Being the foremost consumer and producer of traditional tobacco products worldwide^19^, China also boasts a substantial proportion of electronic cigarette users. As of 2021, an awareness of electronic cigarettes was reported by 86.6% of middle school students and 90.3% of university students in China. Of these students, 16.1% from middle schools and 10.1% from universities confirmed usage of electronic cigarette products^20^. In November 2022, the Chinese government implemented an excise tax on e-cigarette products, signifying a noteworthy transformation in the regulatory landscape of the industry^21^. However, the success of e-cigarette taxation policies is ultimately dependent on the extent to which the industry passes on tax increases and uses strategies to mitigate their impact. Research into industry price-based responses to tax increases is a relatively recent domain in academic inquiry, with a predominant focus on specific countries^4,5,22^. Limited knowledge exists on this matter in the context of China. The introduction of the excise tax on e-cigarettes in 2022 provides a unique opportunity to scrutinize the industry’s responses to this fiscal adjustment.

In October 2022, a joint announcement was issued by the Ministry of Finance, the General Administration of Customs, and the State Taxation Administration, detailing the implementation of an excise tax on e-cigarettes. According to this declaration, effective from 1 November 2022, e-cigarettes would be subjected to an ad valorem tax (VAT), with rates set at 36% for production (or import) and 11% for wholesale transactions^21^. This marks as significant development in the context of China, bringing them in line with the tax rates applicable to conventional cigarettes (those priced at less than 7 RMB per pack). It is essential to note, however, that the current tax system for e-cigarettes in China lacks a ‘specific tax’ component. The comprehensive structure of the Chinese e-cigarette tax structure is detailed in Table 1. This study delves into the industry’s pricing strategies following the introduction of the excise tax, facilitating a thorough assessment of the subsequent impact on market dynamics and the government’s revenue streams. Understanding these strategies is essential for policymakers to develop appropriate countermeasures and hence effective tobacco taxation policies in the future.

**Table 1:** China’s e-cigarette tax structure post-excise implementation in November 2022

*Tax type*	*Tax rate*		*Tax base*
Value-added tax (VAT)	13%		Prices at each segment
Excise	Production level	36%	Production price
	Wholesale level	11%	Wholesale price
Urban maintenance and construction tax and extra charges of educational fee (Cn)	12%		Tax amount of VAT and excise at each segment

## METHODS

In this study, we developed a TaXSiM model tailored to analyze the dynamics of e-cigarettes in China, aligning with the prevailing e-cigarette tax structure and incorporating relevant market statistics. Our approach involved leveraging market data obtained from a representative product, the RELX Phantom Series, to ensure the model’s effectiveness and relevance.

The WHO TaXSiM is designed to describe the current market and tax situation for domestically consumed cigarettes within a particular country or tax jurisdiction. Its purpose extends to projecting the repercussions of tax adjustments on final consumer prices, annual consumption volumes, and government tax revenues. A notable strength of the model lies in its meticulous examination of these outcomes on a brand-specific basis^23^.

**Figure 1:**
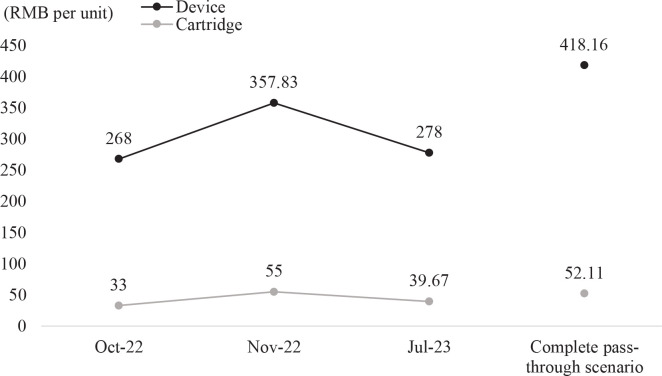
Retail price dynamics of RELX Phantom series pre- and post-excise implementation, China, October 2022 - July 2023

Following this, we performed a policy simulation to thoroughly examine the potential ramifications of introducing an excise tax on e-cigarettes. Our assessment involves scrutinizing the impact of tax increases on prices by observing the dynamic shifts in e-cigarette pricing over time (Figure 1). This approach allows us to gain insights into the industry’s pricing strategies in response to tax adjustments. Additionally, we evaluate the effects of tax adjustments on e-cigarette sales volumes, government tax revenue, industry profits, and other pertinent factors. The data populated into TaXSiM is detailed in Table 2.

**Table 2:** The market data of RELX Phantom series populated into the e-cigarette TaXSiM China model, 2021–2023

*Data type*		*Device*	*Cartridge*	*Data source*
Retail price per unit (RMB)	Pre-excise implementation in Oct. 2022	268	33	Sourced from http://www.relx.fund/cp/165.html
	Post-excise implementation	357.83	55	Sourced in Nov. 2022 from https://ecig.cn/login
		278	39.67	Collected in July 2023 through field investigations conducted in Beijing
Gross profit margin (%)	Wholesale level	36.00	44.00	Sourced in 2021 from https://www.hellohnb.com/
	Retail level	29.60	19.80	Sourced in 2021 from https://www.bluehole.com.cn
RELX’s sales volume in base year of 2021 (million units)		19.50	505.50	Sourced from https://www.hellohnb.com/Cigarette/9025.html
Price elasticity of demand		-0.40		Mao and Hu^26^
RELX’s market share of domestic market in base year of 2021 (%)		62.60		Sourced from https://www.sec.gov/Archives/edgar/data/1828365/000104746921000095/a2242770zf-1a.htm

## RESULTS

### Tax per unit products

Following the implementation of the excise tax on e-cigarettes, there is a notable increase in the applied tax for each e-cigarette device and cartridge, rising from pre-tax levels of 34.53 RMB and 4.25 RMB to 184.69 RMB and 23.36 RMB, respectively. This amounts to a proportional escalation in the tax per unit of 434.87% and 449.65%, respectively.

### Prices per unit under complete pass-through scenario

Before the implementation of the excise tax, the retail prices of e-cigarette devices and cartridges, inclusive of value-added tax (VAT), stood at 268 RMB per unit and 33 RMB per unit, respectively. Assuming the complete transfer of the additional tax to retail prices while maintaining constant gross profit margins throughout the supply chain, the post-tax retail prices for e-cigarette devices and cartridges are anticipated to increase to 418.16 RMB per unit and 52.11 RMB per unit, respectively. This would represent a surge in retail prices by 56.03% and 57.91%, respectively. In a scenario of complete pass-through, the percentage of taxes to retail prices for e-cigarette devices and cartridges would rise from the initial 12.88% to 44.17% and 44.83%, respectively (Table 3).

**Table 3:** Tax and retail price dynamics of RELX Phantom series pre- and post-excise implementation, China, October 2022 – July 2023

			*Device*	*Cartridge*
**Tax per unit (RMB)**	Tax amount pre-excise implementation		34.53	4.25
	Post-excise implementation	Tax amount	184.69	23.36
		Δ tax amount	150.16	19.11
		% of the change in tax	434.87	449.65
**Retail price per unit (RMB)**	Retail price pre-excise implementation		268	33
	Complete pass-through scenario	Retail price	418.16	52.11
		Δ price compared to pre-excise implementation	150.16	19.11
		% of the price change compared to pre-excise implementation	56.03	57.91
	Razor blade model scenario (based on Nov. 2022 price data)	Retail price	357.83	55
		Δ price compared to pre-excise implementation	89.83	22
		% of the price change compared to pre-excise implementation	33.52	66.67
		Δ price compared with complete pass-through scenario	-60.32	2.89
		% of the change in price compared with complete pass-through scenario	-14.43	5.55
	Comprehensive under-shifting scenario (based on July 2023 price data)	Retail prices	278	39.67
		Δ price compared to pre-excise implementation	10	6.67
		% of the change in price compared to pre-excise implementation	3.73	20.21
		Δ price compared with complete pass-through scenario	-140.15	-12.44
		% of the change in price compared with complete pass-through scenario	-33.52	-23.87

### Industry pricing strategies

Right after the enforcement of the excise tax, e-cigarette manufacturers, such as RELX, have strategically adjusted their product pricing. According to data sourced from the National Unified Electronic Cigarette Transaction Management Platform, the retail prices for RELX Phantom Series devices and cartridges in November 2022 rose to 357.83 RMB and 55 RMB, respectively. This reflects a rise of 89.83 RMB for the device and 22 RMB for the cartridges compared to their prices before the tax implementation. Notably, the price increase for the devices was proportionally lower than the implemented tax rate, while the cartridge prices exceeded the tax increment. Therefore, it can be deduced that following the post-tax increase, RELX adopted a pricing strategy reminiscent of the ‘razor blade model’^18^. In implementing this approach, the company chose to under-shift the tax by 60.32 RMB per device, while simultaneously over-shifting by 2.89 RMB per cartridge (Table 3).

The industry’s pricing strategy has undergone adjustments over time. As of July 2023, the RELX Phantom Series was priced at 278 RMB per device and 39.67 RMB per cartridge in retail outlets, as illustrated in Table 3. These figures denote a reduction of 79.83 RMB and 15.33 RMB per unit for e-cigarette devices and cartridges, respectively, in comparison with the prices observed in November 2022. This suggests that RELX, to expand its market share, implemented a comprehensive under-shifting strategy by further lowering prices for both devices and cartridges.

### Simulation strategy to assess effects of excise policy

In 2021, the RELX brand reported the sale of 19.5 million e-cigarette devices and 505.5 million e-cigarette cartridges. Given RELX’s projected market share of 62.6% within the Chinese e-cigarette market in 2020^24^, the total industry sales for devices and cartridges in 2021 could be approximated at 31.15 million and 807.51 million units, respectively.

Due to the absence of empirical research on the price elasticity of demand for e-cigarettes in China, we rely on the widely acknowledged price elasticity of demand for traditional cigarettes, typically estimated to be around -0.4^25,26^ to simulate the excise tax implementation on e-cigarette consumption. Besides, given that the Razor blade model was adopted by RELX for a short while and the comprehensive under-shifting strategy was eventually adopted, we conducted a simulation to assess the impact of excise implementation on e-cigarette sales volume and the government’s revenue under the comprehensive under-shifting scenario which assumes the retail prices of the RELX Phantom series remain constant, similar to the price in July 2023.

### Impact on e-cigarette sales volume

Under the comprehensive under-shifting scenario, it would likely result in a decrease in industry-wide sales. Specifically, we project a decline in e-cigarette device sales to approximately 30.69 million units in 2023. This reflects a decrease of 1.49% compared to 2021. Simultaneously, we project a decline in e-cigarette cartridge sales to roughly 742.25 million units, indicating a decrease of 8.08% compared to 2021 (Table 4).

**Table 4:** Impact on consumption in industry-wide and government’s revenue pre- and post-excise implementation, China, 2021–2023

			*Device*	*Cartridge*	*Sum*
**Sales volume (million units)**	Sales volume pre-excise implementation in 2021		31.15	807.51	
	Complete pass-through scenario	Sales volume	24.17	620.45	
		Δ sales volume compared with the sales volume in pre-excise implementation	-6.98	-187.06	
		% of the change in sales volume compared with the sales volume in pre-excise implementation	-22.41	-23.17	
	Comprehensive under-shifting scenario	Sales volume	30.69	742.25	
		Δ sales volume compared with the sales volume in pre-excise implementation	-0.46	-65.26	
		% of the change in sales volume compared with the sales volume in pre-excise implementation	-1.49	-8.08	
		Δ sales volume compared with complete pass-through scenario	6.52	121.8	
		% of the change in sales volume compared with complete pass-through scenario	26.98	19.63	
**Tax revenue in 2023 (billion RMB)**	Tax revenue pre-excise implementation in 2021		1.08	3.43	4.51
	Complete pass-through scenario	Tax revenue	4.46	14.50	18.96
		Δ tax revenue compared to pre-excise implementation	3.39	11.06	14.45
		% of the change in tax revenue compared to pre-excise implementation	314.87	322.10	320.38
	Comprehensive under-shifting scenario	Tax revenue	5.67	17.34	23.01
		Δ tax revenue compared to pre-excise implementation	4.59	13.91	18.50
		% of the change in tax revenue compared to pre-excise implementation	426.67	404.98	410.16
		Δ tax revenue compared with complete pass-through scenario	1.21	2.84	4.05
		% of the change in tax revenue compared with complete pass-through scenario	27.13	19.59	21.36

### Impact on government’s revenue

Under the comprehensive under-shifting scenario, it is projected that the industry will yield a total of 23.01 billion RMB in turnover taxes for the year 2023. This constitutes a substantial climb of 18.5 billion RMB, which represents an impressive growth of 410.16% in comparison to the untaxed year of 2021, as shown in Table 4.

### Impact on the e-cigarette industry

The comprehensive under-shifting strategy had a significant effect on the industry’s profitability. Highlighting the brand RELX Phantom, for instance, resulted in a net profit reduction of 140 RMB per device and 12 RMB per cartridge, compared to profit levels before the application of excise tax. The intensity of this profit decline undoubtedly expedites the restructuring within the e-cigarette industry, compelling several smaller brands to leave the market rapidly.

## DISCUSSION

The primary goal of this study was to gain insights into the comprehensive pricing strategies adopted by China’s e-cigarette industry, particularly by observing RELX’s responses to taxation. Overall, RELX initially adopted the ‘razor blade model’ immediately after the implementation of the excise tax and eventually employed a ‘comprehensive under-shifting strategy’ several months after the excise tax introduction. Our focus was on determining whether these strategies compromise the effectiveness of the excise tax from a public health perspective, while also assessing their implications on government revenue and the e-cigarette industry.

Under the complete pass-through scenario, it is projected that electronic cigarette product prices would significantly increase, specifically by 56.03% for devices and 57.91% for cartridges, respectively. This anticipated increase in prices might then lead to a decrease in sales volume by approximately 23%, and consequently, a substantial increase in tax revenue by an estimated 320%.

Due to the pricing strategy applied by the tobacco industry, taking the comprehensive under-shifting eventually employed by RELX as an example, the net effect in sales of devices and cartridges was 27% and 20% smaller, respectively, than the full pass-through scenario projections, which suggests that the public health impact of imposing excise taxes on e-cigarettes have been substantially mitigated due to tobacco industry’s under-shifting strategy.

However, this pricing method amplified the fiscal implications. Industry tax revenue from turnover is projected to witness an increase of 21% in 2023 compared to the full pass-through scenario projections.

Notwithstanding the diminished net impact on consumption resulting from the industry’s under-shifting price strategy, the introduction of excise taxes on electronic cigarettes in China in 2022 remains a commendable policy initiative. This measure not only contributes to fiscal revenue but also yields health benefits, establishing a ‘win-win’ scenario.

Nevertheless, to guarantee the intended impact of excise tax increases on pricing effectiveness, Chinese governments and policymakers must proactively monitor the dynamic responses of the e-cigarette industry to tax adjustments and adopt responsive measures. To thwart potential under-shifting strategies employed by the industry to circumvent the intended deterrent effect of tax policies on e-cigarette usage, a prudent step would be to shift the imposition of excise taxes on electronic cigarettes to the retail stage. This strategic adjustment should be complemented by the introduction of an additional specific tax, thoughtfully designed to underscore the health-related benefits associated with the excise taxation on electronic cigarettes. This comprehensive approach aims to optimize the influence of tax policies on both industry behavior and public health outcomes.

### Strengths and limitations

This study marks a pioneering effort in delving into the responses of the Chinese e-cigarette industry to excise adjustments and consequently, their influence on market dynamics and governmental revenue streams. We have devised a specialized TaXSiM model designed exclusively for e-cigarettes in China, intricately incorporating the country’s e-cigarette tax framework. A distinctive advantage of our model is its meticulous scrutiny of these outcomes on a brand-specific level, empowering us to dissect the cumulative impact of the e-cigarette industry’s pricing strategies in response to excise adjustments.

Our study has two principal limitations that warrant acknowledgment. Firstly, our reliance on retail prices derived from field investigations conducted in Beijing introduces a constraint, as these prices may not capture the nationwide variations. It is crucial to recognize that retail prices can exhibit considerable divergence on a broader scale.

Secondly, the imposition of price elasticity into the TaXSiM model constitutes another limitation. Given the absence of empirical research on the price elasticity of demand for e-cigarettes in China, we have opted for the widely accepted price elasticity of demand for traditional cigarettes, typically estimated to be around -0.4. This choice is made to assess the overall impact of excise policies and the industry's reaction on e-cigarette consumption and government revenue. However, it is imperative to acknowledge that this elasticity value may not precisely mirror the authentic demand price elasticity of e-cigarettes in China. This underscores the imperative for further investigation into this aspect to refine our understanding.

## CONCLUSIONS

This study delves into the tobacco industry’s pricing strategies following the introduction of the excise tax, facilitating a thorough assessment of the subsequent impact on market dynamics and the government’s revenue streams. Understanding these strategies is essential for policymakers to develop appropriate countermeasures and hence effective tobacco taxation policies in the future.

## Data Availability

The data supporting this research are available from the authors on reasonable request.
